# Association of Current Opioid Use With Serious Adverse Events Among Older Adult Survivors of Breast Cancer

**DOI:** 10.1001/jamanetworkopen.2020.16858

**Published:** 2020-09-15

**Authors:** Aaron N. Winn, Devon K. Check, Amy Farkas, Nicole M. Fergestrom, Joan M. Neuner, Andrew W. Roberts

**Affiliations:** 1 School of Pharmacy, Department of Clinical Sciences, Medical College of Wisconsin, Milwaukee; 2 Department of Population Health Sciences, Duke University School of Medicine, Durham, North Carolina; 3 Division of General Internal Medicine, Department of Medicine, Medical College of Wisconsin, Milwaukee; 4 Center for Advancing Population Science, Medical College of Wisconsin, Milwaukee; 5 Division of General Internal Medicine, Center for Advancing Population Science, Medical College of Wisconsin, Milwaukee; 6 Department of Population Health, University of Kansas Medical Center, Kansas City; 7 Department of Anesthesiology, University of Kansas Medical Center, Kansas City

## Abstract

**Importance:**

National efforts to improve safe opioid prescribing focus on preventing misuse, overdose, and opioid use disorder. This approach overlooks opportunities to better prevent other serious opioid-related harms in complex populations, such as older adult survivors of cancer. Little is known about the rates and risk factors for comprehensive opioid-related harms in this population.

**Objective:**

To determine rates of multiple opioid-related adverse drug events among older adults who survived breast cancer and estimate the risk of these events associated with opioid use in the year after completing cancer treatment.

**Design, Setting, and Participants:**

This retrospective cohort study used 2007 to 2016 Surveillance, Epidemiology and End Results-Medicare data from fee-for-service Medicare beneficiaries with first cancer diagnosis of stage 0 to III breast cancer at age 66 to 90 years from January 1, 2008, through December 31, 2015, who completed active breast cancer treatment. Data were analyzed from October 31, 2019, to June 10, 2020.

**Exposures:**

Repeated daily measure indicating possession of any prescription opioid supply in Medicare Part D prescription claims.

**Main Outcomes and Measures:**

Adjusted risk ratios (aRRs), estimated using modified Poisson generalized estimating equation models, for adverse drug events related to substance misuse (ie, diagnosed opioid abuse, dependence, or poisoning), other adverse drug events associated with opioid use (ie, gastrointestinal events, infections, falls and fractures, or cardiovascular events), and all-cause hospitalization associated with opioid supply the prior day, controlling for patient characteristics.

**Results:**

Among 38 310 women included in the study (mean [SD] age, 74.3 [6.3] years), there were 0.010 (95% CI, 0.008-0.011) adverse drug events related to substance misuse per 1000 person-days, 0.237 (95% CI, 0.229-0.245) other adverse drug events associated with opioid use per 1000 person-days, and 0.675 (95% CI, 0.662-0.689) all-cause hospitalizations per 1000 person-days. Opioid use was associated with increased risk of adverse drug events related to substance misuse (aRR, 14.62; 95% CI, 9.69-22.05; *P* < .001), other adverse drug events related to opioid use (aRR, 2.50; 95% CI, 2.11-2.96; *P* < .001), and all-cause hospitalization (aRR, 2.77; 95% CI, 2.55-3.02; *P* < .001). In a dose-response effect, individuals with high daily opioid doses had consistently higher risks of all study outcomes compared with individuals who had low opioid doses. Compared with days with no opioid exposure, the risk of any adverse drug event related to substance misuse was 3.4-fold higher for individuals with a current opioid supply ≥50 mg morphine equivalent dose per day (aRR, 3.40; 95% CI, 2.47-4.68; *P* < .001), while the risk was 2.3-fold higher for individuals with 1 to 49 mg morphine equivalent dose per day (aRR, 2.29; 95% CI, 1.89-2.77; *P* < .001).

**Conclusions and Relevance:**

These findings suggest that among older adults who survived breast cancer, continued prescription opioid use in the year after completing active cancer treatment was associated with an immediate increased risk of a broad range of serious adverse drug events related to substance misuse and other adverse drug events associated with opioid use. Clinicians should consider the comprehensive risks of managing cancer pain with long-term opioid therapy.

## Key Points

**Question:**

What are the risks of serious adverse events associated with continued prescription opioid use after completing active treatment for breast cancer?

**Findings:**

In this cohort study among 38 310 older adults who survived breast cancer, opioid use after active cancer treatment was associated with a nearly 15-fold increase in immediate risk of adverse drug events related to substance misuse, including overdose, and a 2- to 3-fold increased immediate risk of other adverse drug events related to opioid use, such as falls and fractures or all-cause hospitalization. Other adverse drug events related to opioid use were more than 20-fold more common than events related to substance misuse.

**Meaning:**

These findings suggest that minimizing unnecessary long-term opioid therapy after active cancer treatment may prevent a broad range of avoidable opioid-related harms, including those related and unrelated to substance misuse.

## Teaser

This cohort study examines rates and estimated risk of adverse drug events associated with opioid use in the year after cancer treatment among older adults who survived breast cancer.

## Introduction

The primary focus of opioid policies and prescribing guidelines^1^ that have emerged in response to the opioid crisis is to prevent misuse, overdose, and development of opioid use disorder. However, efforts to reduce unsafe opioid use may also prevent other serious opioid-related adverse drug events, including gastrointestinal (GI) complications, cardiac events, infection, and falls and fractures associated with sedation and impaired cognition.^2^

Preventing serious opioid harms not related to substance misuse may be particularly urgent for certain at-risk populations, including older adults who have survived cancer. Older adults, who account for nearly two-thirds of the 16 million people who have survived cancer^3^ in the US, have high rates of opioid use^4,5^ for cancer pain as well as significant clinical complexities owing to their age,^6^ comorbid conditions,^3,7^ and cancer treatments. These characteristics may predispose this population to adverse drug events not related to opioid misuse.

However, little is known about the association between active opioid therapy continuing after active cancer treatment and comprehensive opioid-related serious adverse drug events. The purpose of this study was to examine the risk of a broad range of opioid-related serious adverse drug events among older individuals who survived breast cancer, with the goal of informing tailored opioid prescribing recommendations for this large, at-risk, and overlooked population.

## Methods

### Data and Cohort

This retrospective cohort study was approved and determined exempt from informed consent by the University of Kansas Medical Center institutional review board because data were deidentified and study investigators had no direct or indirect access to personally identifiable information. We adhered to the Strengthening the Reporting of Observational Studies in Epidemiology (STROBE) reporting guideline for observational cohort studies. The study used Surveillance, Epidemiology and End Results-Medicare data for 2007 to 2016 to estimate the immediate risk of opioid-related adverse drug events associated with time-dependent daily opioid exposure in the year after active breast cancer treatment. We focused on this period to examine harms from potentially avoidable long-term opioid therapy extending beyond the active cancer treatment phase.^8^ We defined the end of active cancer treatment as the last day following the breast cancer diagnosis date on which an individual was recorded as receiving chemotherapy, radiation, or curative surgical cancer treatment.^9^

We evaluated women aged 66 to 90 years diagnosed with Surveillance, Epidemiology and End Results-confirmed stage 0 to III breast cancer from January 1, 2008, to December 31, 2015, who did not have a previous cancer diagnosis. We required women to have continuous Medicare Parts A and B coverage from 12 months before their breast cancer diagnosis through 12 months after the end of active breast cancer treatment; we also required women to have Medicare Part D coverage from 3 months before diagnosis through 12 months after the end of active breast cancer treatment. We excluded individuals who died or recorded a second primary cancer diagnosis during the 12 months of follow-up after diagnosis; did not have a full 12 months of follow-up before December 31, 2016; or had any prior opioid prescription filled in the 3 months before diagnosis.

### Measures

Outcome measures included time-dependent daily binary measures indicating whether an individual experienced a serious opioid-related adverse drug event on a given day during the 12-month follow-up period after the individual had completed active cancer treatment. Opioid-related adverse drug events consisted of those related to substance misuse (ie, diagnosed opioid dependence, abuse, or poisoning)^10^ and those unrelated to substance misuse or opioids’ abuse or overdose potential (ie, serious infection,^11^ GI events,^12^ falls and fractures,^12^ cardiovascular events,^12^ and all-cause hospitalization) (eFigure in the Supplement). Days when an individual was hospitalized were excluded from analyses.

The primary independent variable was a time-dependent binary measure indicating exposure vs no exposure to prescription opioids on each day across the 12-month follow-up period, using Part D prescription fill data. Time-invariant covariates included cancer diagnosis, treatment characteristics, and additional patient demographic and clinical characteristics measured during the 12-month baseline period before cancer diagnosis.

### Statistical Analysis

Descriptive statistics reported outcome, demographic, clinical, and treatment characteristics for the cohort. We estimated the unadjusted rate of each outcome event per 1000 person-days in the year after active breast cancer treatment. We leveraged our time-dependent daily outcome and exposure measures using modified Poisson generalized estimating equations^13^ to estimate adjusted risk ratios (aRRs) for all adverse drug event outcomes associated with opioid exposure on the preceding day compared with no opioid exposure on the previous day. We lagged opioid exposure by 1 day to protect against erroneous conclusions related to reverse causation. Generalized estimating equations did not converge for the substance misuse–related event outcome, so we accounted for repeated daily observations for this outcome by clustering SEs within individuals. We adjusted for time-invariant patient demographic, clinical, cancer diagnosis, and cancer treatment characteristics.

The data were analyzed from October 31, 2019, to June 10, 2020. All analyses were performed using 2-sided hypothesis tests in Stata statistical software version 16 (StataCorp), and significance was assumed as *P* < .05.

We conducted sensitivity analyses to assess for a dose-response association between the intensity of an individual’s current opioid exposure and risk of a serious opioid-related adverse drug event. In this analysis, we characterized daily opioid exposure measures as high (≥50 mg morphine equivalent dose [MED] per day), low (1-49 mg MED per day), or none.^14^ We also measured opioid exposure intensity as a rolling mean of daily milligrams of MED over the previous 14 days, dichotomized as high vs low opioid exposure.

## Results

We analyzed a cohort of 38 310 older women with mean (SD) age 74.3 (6.3) years (Table 1). There were 0.010 (95% CI, 0.008-0.011) adverse drug events related to substance misuse per 1000 person-days, 0.237 (95% CI, 0.229-0.245) other adverse drug events associated with opioid use per 1000 person-days, and 0.675 (95% CI, 0.662-0.689) all-cause hospitalizations per 1000 person-days (Table 2). Other adverse drug events associated with opioid use were 23.7-fold more common than adverse drug events related to opioid misuse. Our adjusted analysis showed a consistent positive association between current opioid exposure and serious adverse drug events (Figure). Compared with days without opioid exposure, current opioid exposure was associated with 2.5-fold higher risk of a serious adverse drug event not related to substance misuse (aRR, 2.50; 95% CI, 2.11-2.96; *P* < .001) and nearly 3-fold the immediate risk of all-cause hospitalization (aRR, 2.77; 95%, CI, 2.55-3.02; *P* < .001).

**Table 1.** Patient Characteristics

Characteristic	No. (%) (N = 38 310)
Age, y	
Mean (SD)	74.7 (6.3)
66-70	12 231 (31.9)
71-75	10 449 (27.3)
76-80	7884 (20.6)
81-85	5233 (13.7)
86-90	2513 (6.7)
Race/ethnicity	
White	31 481 (82.2)
Black	2401 (6.3)
Hispanic	2072 (5.4)
Other	2356 (6.2)
Low-income subsidy	7752 (20.2)
Charlson comorbidity score	
0	20 332 (53.1)
1	10 471 (27.3)
≥2	7507 (19.6)
Stage	
0	7113 (18.6)
I	18 872 (49.3)
II	9967 (26.0)
III	2358 (6.2)
Tumor size, cm	
≤2	25 337 (66.2)
>2 to <5	8938 (23.3)
≥5	1827 (4.8)
Unknown	2168 (5.7)
Breast surgical treatment	
Mastectomy	
Double	1266 (33.1)
Partial	22 773 (59.4)
Lymph node surgical treatment	1145 (3.0)
Tumor biopsy	1525 (4.0)
None	198 (0.5)
Hormonal therapy	11 312 (29.5)
Radiation	20 720 (54.1)
Adjuvant chemotherapy	5228 (13.7)
Any chemotherapy	6213 (16.2)
Trastuzumab	1650 (4.3)
Taxane	5452 (14.2)
Doxorubicin	1710 (4.5)

**Table 2.** Unadjusted Outcome Event Rates per 1000 Patient-Days

Outcome	No.					
	Total		With current opioid exposure		Without current opioid exposure	
	Events	Per 1000 patient-days (95% CI)	Events	Per 1000 patient-days (95% CI)	Events	Per 1000 patient-days (95% CI)
Days at risk	13 666 612	NA	373 272	NA	13 293 340	NA
Substance misuse–related ADE^a^	130	0.010 (0.008-0.011)	45	0.121 (0.085-0.156)	85	0.006 (0.005-0.008)
Other opioid use–associated ADE^b^	3242	0.237 (0.229-0.245)	328	0.879 (0.784-0.974)	2914	0.219 (0.211-0.227)
GI event	322	0.024 (0.021-0.026)	31	0.083 (0.054-0.112)	291	0.022 (0.019-0.024)
Fall or fracture	1329	0.097 (0.092-0.103)	133	0.356 (0.296-0.417)	1196	0.090 (0.085-0.095)
Cardiovascular event	710	0.052 (0.048-0.056)	63	0.169 (0.127-0.211)	647	0.049 (0.045-0.052)
Serious infection	927	0.068 (0.064-0.072)	106	0.284 (0.230-0.338)	821	0.062 (0.058-0.066)
All-cause hospitalization	9228	0.675 (0.662-0.689)	953	2.553 (2.391-2.715)	8275	0.623 (0.609-0.636)

Abbreviations: ADE, adverse drug event; GI, gastrointestinal; NA, not applicable.

^a^ Includes diagnosed opioid abuse, dependence, and poisoning.

^b^ May be smaller than the separate total number of events if an individual is diagnosed with more than 1 adverse drug event during a single health care encounter.

**Figure.**
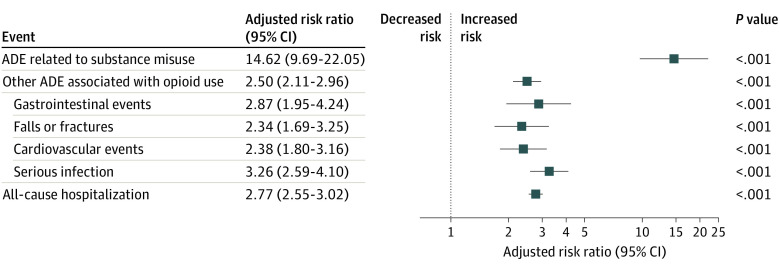
Adverse Drug Events (ADEs) Associated With Current Opioid Use vs No Current Opioid Use After Breast Cancer Treatment Point estimates (boxes) and 95% CIs (whiskers) were estimated using modified Poisson generalized estimating equations for the association between any current opioid exposure vs no current opioid exposure on ADE outcomes in the year after completing active breast cancer treatment. The substance misuse–related ADE model was estimated using modified Poisson clustering on individual because of lack of convergence in generalized estimating equations. Estimates adjusted for age, race/ethnicity (ie, White, Black, Hispanic, or other), low-income subsidy receipt, comorbidity score (ie, 0, 1, or ≥2), cancer stage (ie, 0, I, II, or III), tumor size (ie, ≤2 cm, <2 to <5 cm, ≥5 cm, or unknown size), breast surgical treatment (ie, mastectomy, partial mastectomy, lymph node surgery, tumor biopsy, or none), use of hormonal therapy, use of radiation, use of adjuvant chemotherapy, use of any chemotherapy, any use of trastuzumab, any use of a taxane, and any use of doxorubicin. Vertical dotted line indicates reference null adjusted risk ratio of 1.00.

Opioid exposure was also associated with higher immediate risk of an adverse drug event related to substance misuse (aRR, 14.62; 95% CI, 9.69-22.05; *P* < .001). Among specific other serious adverse drug events related to opioid use, current opioid exposure was associated with increased risk of GI events (aRR, 2.87; 95% CI, 1.95-4.24; *P* < .001), falls and fractures (aRR, 2.34; 95% CI, 1.69-3.25; *P* < .001), cardiovascular events (aRR, 2.38; 95% CI, 1.80-3.16; *P* < .001), and serious infections (aRR, 3.26; 95% CI, 2.59-4.10; *P* < .001). In sensitivity analyses, we found a consistent dose-response association between opioid exposure intensity and serious adverse drug events (eFigure in the Supplement). For example, compared with days with no opioid exposure, the risk of any adverse drug event related to substance misuse was 3.4-fold higher for individuals with a current opioid supply ≥50 mg MED per day (aRR, 3.40; 95% CI, 2.47-4.68; *P* < .001), while the risk was 2.3-fold higher for individuals with 1 to 49 mg MED per day (aRR, 2.29; 95% CI, 1.89-2.77; *P* < .001).

## Discussion

In this cohort study of older adults who survived breast cancer, we found that individuals with current opioid exposure, especially high-dose opioid exposure, on a given day in the year after active breast cancer treatment had significantly higher immediate adjusted risk of myriad serious adverse drug events compared with their risk on days without opioid exposure. Notably, the risks of GI events, infection, falls and fractures, and cardiovascular events were 2- to 3-fold higher on days with current opioid exposure. These serious adverse drug events were also more than 20-fold more common than events related to substance misuse, such as opioid overdose. As efforts to increase opioid safety for treating cancer pain increase, it is important for clinicians to recognize that opioid-related risks in this population extend beyond misuse, overdose, and opioid use disorder.

### Limitations

This study has some limitations. First, prescription fill records in claims data do not equate to use. Second, we examined a 1-day lag in association between opioid supply and adverse drug events, so as not to exclude individuals hospitalized for an adverse drug event. Because of this, we could not assess instances in which individuals received new opioid therapy earlier in the same day that they recorded an adverse drug event. Third, opioids are often used as needed, meaning individuals may use the medications beyond the period accounted for by the days’ supply in the Part D claim. Our sensitivity analysis used a 14-day rolling mean and produced similar results to analyzing the minimum day’s amount. Fourth, this study may include confounding by indication, in which individuals with more severe symptoms receive more opioid therapy. We believe that a broad outcome, such as all-cause hospitalization, may be subject to this bias, but the adverse drug events related to substance misuse and other adverse drug events related to opioid use (eg, GI or cardiovascular events) are less likely to have such a bias. Fifth, our findings may not generalize to other cancers or younger populations.

## Conclusions

Opioids remain a mainstay of cancer pain management; however, increasing evidence indicates that the benefits of long-term opioid use for cancer pain may be limited.^15^ Our findings add to these concerns by demonstrating that opioid therapy significantly increases the risk of multiple preventable serious adverse drug events. Clinicians should carefully weigh the benefits and comprehensive risks of opioid therapy in older adult survivors of breast cancer who have completed active breast cancer treatment.

## Notes

Supplement. **eFigure.** Adjusted Risks of Potentially Avoidable Adverse Events Associated With High- and Low-Dose Current Opioid Use vs No Current Opioid Use After Completing Active Breast Cancer Treatment
