# Supplementary material for: Association of Current Opioid Use With Serious Adverse Events Among Older Adult Survivors of Breast Cancer
Source: JAMA Netw Open. 2020 Sep 15;3(9):e2016858. doi: 10.1001/jamanetworkopen.2020.16858 (PMC7492912; doi:10.1001/jamanetworkopen.2020.16858)
Supplement: Supplement. — eFigure. Adjusted Risks of Potentially Avoidable Adverse Events Associated With High- and Low-Dose Current Opioid Use vs No Current Opioid Use After Completing Active Breast Cancer Treatment [file jamanetwopen-e2016858-s001.pdf]

Supplementary Online Content

Winn AN, Check DK, Farkas A, Fergestrom NM, Neuner JM, Roberts AW. Association of current opioid use with serious adverse events among older adult survivors of breast cancer. *JAMA Netw Open*. 2020;3(9):e2016858. doi:10.1001/jamanetworkopen.2020.16858

eFigure. Adjusted Risks of Potentially Avoidable Adverse Events Associated With High- and Low-Dose Current Opioid Use vs No Current Opioid Use After Completing Active Breast Cancer Treatment

This supplementary material has been provided by the authors to give readers additional information about their work.

© 2020 Winn AN et al. *JAMA Network Open.*

eFigure. Adjusted Risks of Potentially Avoidable Adverse Events Associated With High- and Low-Dose Current Opioid Use vs No Current Opioid Use After Completing Active Breast Cancer Treatment

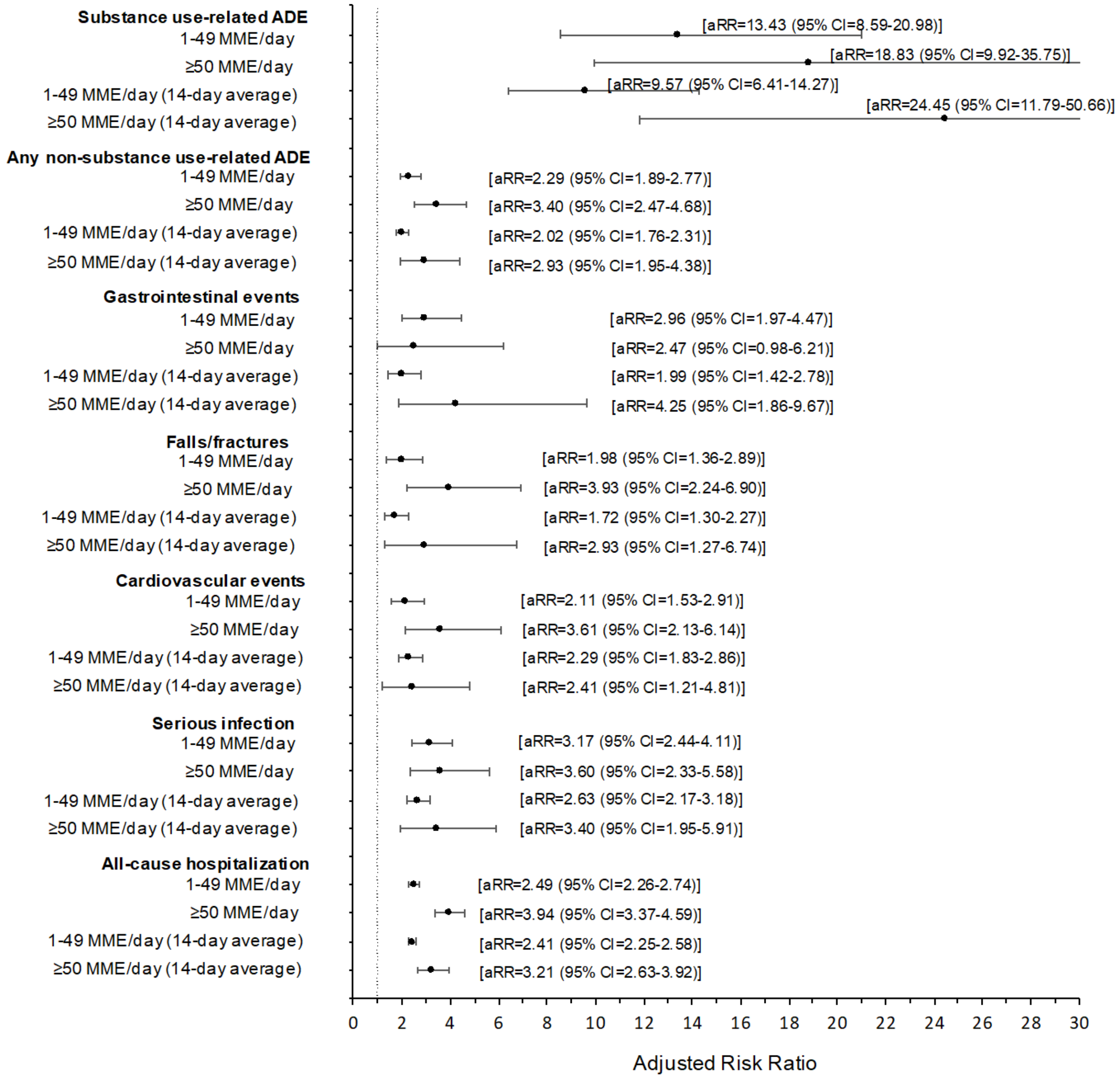

Note: ADE=Adverse drug event; aRR=Adjusted risk ratio; MME=Morphine milligram equivalents; CI=confidence interval. The vertical dotted line indicates the reference null aRR of 1.0. Point estimates and 95% confidence intervals were estimated using modified Poisson generalized estimating equations for the association of any current high-dose opioid exposure (≥50 MME/day) and low-dose current opioid exposure (1-49 MME/day) versus no current opioid exposure with ADE outcomes in the year after completing active breast cancer treatment. Substance use-related ADE model was estimated using modified Poisson clustering on individual because of lack of convergence in GEE. Above estimates were adjusted for age, race (white, black, Hispanic, other), low-income subsidy receipt, comorbidity score (0, 1, 2+), stage (0, I, II, III), tumor size in centimeters (≤2, <2 to <5, 5+, unknown size), breast surgery (mastectomy, partial mastectomy, lymph node surgery, tumor biopsy, and no surgery), use of hormonal therapy, use of radiation, use of adjuvant chemotherapy, use of any chemotherapy, any use of trastuzumab, any use of a taxane, and any use of doxorubicin.

© 2020 Winn AN et al. *JAMA Network Open.*
